# CopomuS—Ranking Compensatory Mutations to Guide RNA-RNA Interaction Verification Experiments

**DOI:** 10.3390/ijms21113852

**Published:** 2020-05-28

**Authors:** Martin Raden, Fabio Gutmann, Michael Uhl, Rolf Backofen

**Affiliations:** 1 Bioinformatics, Department of Computer Science, University Freiburg, Georges-Koehler-Allee 106, 79110 Freiburg, Germany; fabio.gutmann@jupiter.uni-freiburg.de (F.G.); uhlm@informatik.uni-freiburg.de (M.U.); backofen@informatik.uni-freiburg.de (R.B.); 2 Signalling Research Centres BIOSS and CIBSS, University of Freiburg, Schaenzlestr. 18, 79104 Freiburg, Germany

**Keywords:** RNA-RNA interaction, compensatory mutation, mutation, design, sRNA

## Abstract

In silico RNA-RNA interaction prediction is widely applied to identify putative interaction partners and to assess interaction details in base pair resolution. To verify specific interactions, in vitro evidence can be obtained via compensatory mutation experiments. Unfortunately, the selection of compensatory mutations is non-trivial and typically based on subjective ad hoc decisions. To support the decision process, we introduce our COmPensatOry MUtation Selector CopomuS. CopomuS evaluates the effects of mutations on RNA-RNA interaction formation using a set of objective criteria, and outputs a reliable ranking of compensatory mutation candidates. For RNA-RNA interaction assessment, the state-of-the-art IntaRNA prediction tool is applied. We investigate characteristics of successfully verified RNA-RNA interactions from the literature, which guided the design of CopomuS. Finally, we evaluate its performance based on experimentally validated compensatory mutations of prokaryotic sRNAs and their target mRNAs. CopomuS predictions highly agree with known results, making it a valuable tool to support the design of verification experiments for RNA-RNA interactions. It is part of the IntaRNA package and available as stand-alone webserver for ad hoc application.

## 1. Introduction

**Figure 1:**
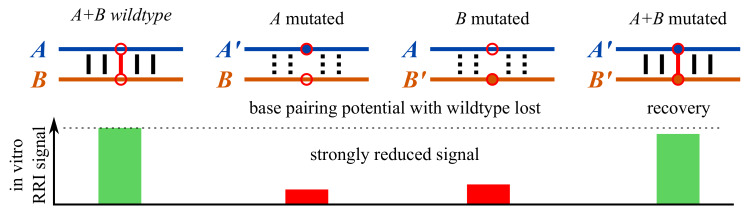
Depiction of an RNA-RNA interaction verification experiment based on compensatory mutations of two RNA sequences *A* and *B*. The mutated nucleotides are highlighted by red circles. The lost and regained base pair is given as red line. Black solid lines depict likely formed RRI base pairs, while unlikely base pairs (instable due to reduced RRI) are represented in dotted lines.

Many non-coding (nc)RNAs, like bacterial small (s)RNAs or eukayotic micro (mi)RNAs, perform their regulatory functions via direct RNA-RNA interaction (RRI) with their target RNAs [1]. This allows the use of in silico RRI prediction approaches to identify potential targets [2,3], which even provide interaction models in base pair resolution. To verify such predictions in vitro, mutation or deletion experiments are conducted [3,4]. Within deletion experiments, whole (potentially interacting) subsequences are removed from one or both RNAs and the in vitro measured interaction potential is compared to results using wildtype sequences. Since deletion of subsequences can have strong side effects, more sophisticated verification experiments are based on compensatory mutations (CoMs). To this end, both potentially interacting RNAs are mutated, such that wildtype-mutant combinations have a reduced base pairing potential within the interaction site that is regained in mutant-only interactions. Often, only a single base pair is mutated, but multiple concurrent CoMs are also used in literature [5]. A depiction of the setup is given in Figure 1. The in vitro RRI potential can be assessed via various experimental protocols [1], e.g., GFP reporter systems [3]. An RRI is considered verified via CoM if the in vitro RRI signal of the wildtype interaction is lost after mutating one sequence, and recovered when both RNAs are mutated (see Figure 1).

The success of a CoM-based RRI verification strongly depends on the mutation selection. If the base-pair-breaking mutation is not weakening the RRI strong enough, no effect is detectable even if the functional RRI is mutated. On the other hand, if the mutation dramatically changes the intra-molecular structure formation of the respective RNA (e.g. when mutating multiple nucleotides at once), reduced RRI signal might be caused by an inaccessibility of the interaction site rather than the loss of base pairing potential. Thus, the design of potent CoMs is typically done manually based on the personal experience of the experimenter.

Here, we introduce CopomuS, a compensatory mutation selector to support this decision process. CopomuS in silico investigates and compares the effect of CoMs on RRI formation to rank candidate CoMs by their verification potential. To this end, IntaRNA [6,7], a state-of-the-art RRI prediction tool [8], is applied. For each CoM, RRI characteristics of the 4 wildtype-mutant sequence combinations are provided. This annotated list of CoMs is sorted by decreasing verification potential (as assessed by CopomuS) and enables an objective subsequent pruning by the experimenter given his/her expert knowledge. That way, CopomuS very much simplifies CoM selection and reliefs the experimenter of the otherwise necessary manual RRI prediction and comparison. Besides the saving of time, users can easily test the effect of different RRI and CoM constraints on the mutations of interest and the results are simple to reproduce. Finally, CopomuS translates the hypothesis behind compensatory mutation experiments into the respective theoretical model. That is, it implements a meta-strategy that checks for and ensures the intended stability difference pattern of wildtype versus mutant combinations.

## 2. Materials And Methods

### 2.1. Compensatory Mutations From Literature

To develop and evaluate CopomuS, we first extracted CoMs of successfully verified RRIs from literature, focusing on sRNA-target RRIs where sRNA binding inhibits translation of the target mRNA. The data was extracted from the benchmark set introduced in [9] and comprises both single-nucleotide CoMs (Table A1; 31 RNA pairs) as well as CoMs involving multiple concurrently mutated nucleotides (Table A2; 28 RNA pairs). To model the workflow based on an sRNA target prediction e.g., following [2,3], we use a genomic context around the start codon for each target. All sequences are provided in Table A3, Table A4, Table A5 and Table A6.

### 2.2. CopomuS Workflow

The central assumptions of CopomuS are that

(1) the (main) regulatory RRI between the RNAs is defined by a single interaction site not interrupted by intra-molecular base pairing and
(2) IntaRNA’s prediction model correctly identifies the important parts of this regulatory RRI.

Only under Assumption-1 we can expect a single CoM to sufficiently alter the RRI potential between the RNAs, since the loss of one site can not be compensated by a concurrently formed second site. A classic example for such a multi-site RRI is the OxyS-fhlA interaction [10]. Furthermore, only under Assumption-2 CopomuS is expected to provide reasonable CoM candidates, since it generates and evaluates them based on the most stable RRIs predicted by IntaRNA.

#### 2.2.1. CoM Generation

Following Assumption-2, CopomuS first computes the most stable RRI that can be formed by the wildtype RNAs using IntaRNA. The (minimal) free energy (MFE) estimate of the RRI computed by IntaRNA provides a stability proxy to assess the RRI potential. That is, the lower the energy the more stable the formed RRI is. Furthermore, the lowest energy RRI is the most likely when assuming a Boltzmann distribution of the energies [11]. The Nearest-Neighbor-model-based energy estimates incorporate both the stability of the inter-molecular base pairing (hybridization energy) and penalty (ED) terms to reflect the accessibility of the interacting subsequences [7]. More precisely, the latter describes the energy needed to break all intra-molecular base pairs that are formed by the RRI’s subsequences [12].

CopomuS supports two modes to select inter-molecular base pairs for CoM candidate generation. Per default, all base pairs of the MFE RRI are considered. In addition, one can extend this set with all base pairs of suboptimal RRIs that can be formed by the subsequences covered by the MFE RRI. This softens the dependency on the accuracy of the reported MFE RRI base-pair pattern if alternative patterns within the same site are possible.

Next, identified base pairs are further filtered given user-defined constraints. For instance, specific base pair types, like AU or GU base pairs, can be filtered. Furthermore, lonely base pairs that can not stack with another base pair on either side can be excluded, since they typically provide only low stability contributions. Similarly, base pairs at putative ends of inter-molecular helices can be removed, since they can only form stackings on one side.

Finally, given the set of base pairs to be considered for mutation, the user can define whether all possible CoMs per base pair are to be considered (i.e., 3 non-compatible mutation alternatives for a given base pair like e.g., GU, GC, AU for UA (Note, CG is omitted since wildtype U can form a base pair with mutant G, which needs to be prevented)) or if the respective CoM candidate is only the ’nucleotide flip’ of the base pair (as often done in literature), i.e., mutating a GC into a CG base pair.

At the end of the CoM generation, CopomuS outputs a list of CoM candidates to be evaluated and ranked.

#### 2.2.2. CoM Characteristics and Ranking

**Figure 2:**
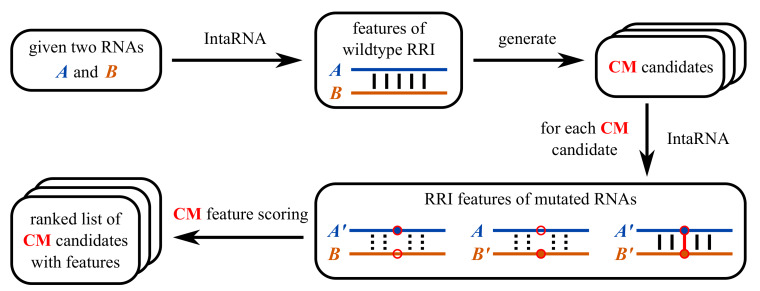
Workflow of CopomuS to generate and rank CoM candidates based on IntaRNA RRI predictions and respective characteristics.

Each CoM candidate is evaluated, using IntaRNA MFE-RRI predictions for all 4 sequence combinations for the current CoM, i.e., wildtype-only, wildtype-mutant as well as mutant-only combinations (see Figure 2). Depending on which ranking the user has specified, various RRI characteristics are aggregated for each combination, e.g., RRI stability in terms of MFE, RRI position, base pairs, or the accessibility of the mutated positions.

CopomuS implements a hierarchical CoM ranking. To this end, different classification and sorting functions are provided that can be sequentially combined to select for specific CoM characteristics. The most important classifier mfeCover checks whether the CoM is also covered by the mutant-only RRI. If not, the mutant-only RRI was found in a different location or no stable RRI was found at all. Next is the E classifier, which evaluates the desired reduction in RRI potential of wildtype-mutant combinations compared to wildtype-only or mutant-only RRIs. As discussed, the RRI MFE provides a proxy to assess interaction stability. Thus, CopomuS demands that both wildtype-only (ww) and mutant-only (mm) MFE are below zero and both wildtype-mutant MFEs are worse (higher), using two thresholds α and β, respectively. More formally, MFE(ww) + α<min(MFE(mw),MFE(wm)) and MFE(mm) + β<min(MFE(mw),MFE(wm)) have to be satisfied.

This way, CopomuS can identify CoM candidates that have a high chance to show the expected in vitro RRI signal pattern from Figure 1 needed for RRI verification. The combination and hierarchy of classifier functions already defines a ranking of CoM subsets with decreasing level of constraint satisfaction.

Finally, each subset can be sorted via the minDeltaE function to get a final ranking of CoM candidates. minDeltaE favors CoMs with higher minimal MFE difference between wildtype-mutant combinations to wildtype-only or mutant-only combinations, i.e., it calculates and compares (min(MFE(mw),MFE(wm))−max(MFE(ww),MFE(mm))). Therefore, the top-ranked CoM candidate will show the strongest RRI stability reduction for both wildtype-mutant combinations.

### 2.3. Availability

CopomuS is implemented in Python and part of the IntaRNA package version 3.2.0 or higher (freely available at https://github.com/BackofenLab/IntaRNA/). Due to its modular implementation, it can be easily expanded by further classification or sorting functions. Given its flexible command line interface and the provided CSV-based output, CopomuS can be easily integrated in automated pipelines and workflows. Its webserver (freely available at http://rna.informatik.uni-freiburg.de/CopomuS/) for ad hoc usage is part of the Freiburg RNA tools framework [13] version 4.8.0 or higher.

## 3. Results

To evaluate the rationals behind CopomuS and to better understand CoM characteristics, we studied RRI characteristics of CoMs from literature. Subsequently, these CoMs were also used to benchmark CopomuS.

### 3.1. Statistics of CoMs from Literature

**Figure 3:**
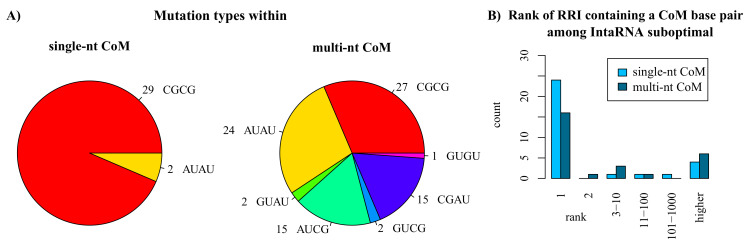
Characteristics of CoMs known from the literature. (**A**) Distribution of mutation types where the first two letters encode the wildtype nucleotides and the last two the respective mutated bases (both lex-sorted to reduce classes). For instance, AUCG represents both an AU or a UA wildtype base pair mutated either to CG or GC; (**B**) Rank of the RRI containing a CoM base pair among IntaRNA’s energy-sorted suboptimal RRI list.

First, the distribution of mutation types was assessed, which is shown in Figure 3A. Within the single-nt CoM data set, mainly GC base pairs (29/31) have been mutated and all base pairs were ’flipped’, i.e., mutated into their wildtype pairing partner. Multi-nt CoMs are also dominated by flipped mutations (52/86) and mutations of GC wildtype base pairs (42/86).

Next, we studied whether or not IntaRNA is able to correctly identify the CoM. We therefore identified the rank of the RRI predicted by IntaRNA (including suboptimals; sorted by energy) that includes at least one of the base pairs of the CoM. Respective statistics are provided in Figure 3B. Most single-nt CoMs are within IntaRNA’s MFE RRI prediction (rank 1; 24/31), while 4 CoMs are not covered by IntaRNA predictions. Similar results are observed for multi-nt CoMs, where 16/27 could be mapped to MFE RRIs and 6/27 are without respective prediction.

All base pairs of the single-nt CoMs can form base pair stackings (to at least one side), i.e., no lonely base pairs have been mutated. Most (24/31) single-nt CoMs are within helices or can stack to both sides.

### 3.2. Energy Profiling of CoMs from Literature

**Figure 4:**
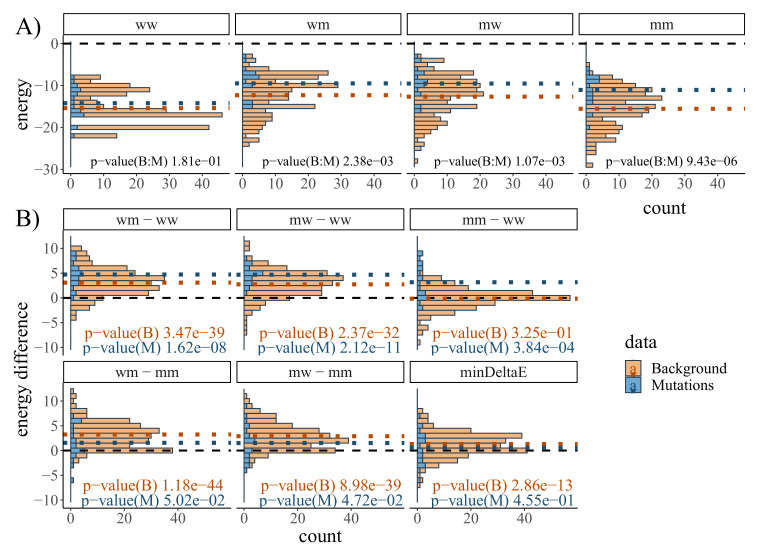
(**A**) Minimum free energy (MFE) distributions of known single-nt GC-mutating CoMs and their background model. The ’Mutations’ data (blue hues) covers 22 CGCG CoMs known from literature, while the ’Background’ data (orange hues) aggregates all remaining 207 CGCG CoMs from the MFE RRIs containing the known CoMs. There are four possible sequence combinations, referring to sRNA-mRNA pairs with respective (w)ildtype/(m)utant annotation. That is, an interaction of wildtype sRNA with the mutated mRNA is denoted by ’wm’, while e.g., ’mm’ refers to the interaction of sRNA and mRNA mutant. Each subplot provides the p-value of the sample t-test comparing the respective distributions. Dotted lines mark mean values, while dashed black lines highlight an energy difference of zero; (**B**) Pairwise energy differences of mutant combinations compared to wildtype-only ’ww’ or mutant-only ’mm’ MFEs for the CoMs of both the Mutations and Background data set. That is, e.g., ’wm-mm’ refers to the energy difference of a ’wm’ interaction and the respective mutant-only ’mm’ interaction energy. For each data set, p-values of paired sample t-tests that compare the values with the respective reference MFEs (’ww’ or ’mm’) are provided. The minDeltaE feature is defined as (min(MFE(mw),MFE(wm))-max(MFE(ww),MFE(mm))). For further details, see text.

Next, we evaluated if IntaRNA MFE predictions are a useful proxy for RRI stability in order to identify highly potent CoM candidates based on the RRI stability differences between wildtype-/mutant-only (ww, mm) and wildtype-mutant combinations (wm, mw). We restricted the investigation to the 22 single-nt GC-mutating CoMs found in MFE RRIs. As a background model, we identified all additional GC base pairs (in total 207) from their respective MFE RRIs and treated them as flipped CGCG mutations. For each CoM and wildtype-mutant combination, we computed the respective MFE. Figure 4 visualizes the MFE distributions as well as MFE differences for both the CoMs known from literature as well as the background CoMs.

First, we compared the energy distributions of known CoMs and the background shown in Figure 4A. That is, for each wildtype-mutant combination (ww, wm, mw, mm), we compare the set of known CoM MFE values with the set of background CoM MFE values. This is done based on a sample t-test (known CoMs vs. background CoMs, two-tailed, unequal variance, unpaired). Respective p-values are reported in Figure 4A. Here, we only find the mutant-only combinations (mm) to be significantly different.

We also compared for each CoM the MFE of a given RNA combination (e.g., wm) with the respective value of the wildtype-only (ww) or mutant-only (mm) combination from the same data set. Beside visualizing the differences, Figure 4B also shows the p-values of respective paired sample t-tests (combination vs. ww|mm, two-tailed, unequal variance). With regard to wildtype-only (ww) MFE, both the known CoMs as well as the background CoMs show on average higher wildtype-mutant energies (i.e., wm-ww, mw-ww are positive). For the known CoMs, this also holds for mutant-only combinations (mm-ww), while the background CoMs do not show a significant difference between wildtype-only and mutant-only energies (mm-ww). That is, known CoMs show on average a stronger reduction of RRI potential of compensatory mutations (mm-ww) compared to the background. This difference also manifests in the energy differences when relating to the MFE of mutant-only (mm) combinations, i.e., the wildtype-mutant combinations of known CoMs (wm-mm, mw-mm) show in contrast to the background model on average no significant energy change. This is also reflected in the minDeltaE distributions, i.e., the mean minDeltaE difference is about zero for known CoMs while a slightly positive average value is observed for the background.

### 3.3. CopomuS Parameter Optimization

To find suitable values for the α/β parameters of the MFE-based CoM classification, we performed a parameter sweep in the range [1,5] given the observations from Figure 4. Here, we used the whole single-nt CoM data set and restricted the CoM candidate generation to the flipping of the MFEs’ GC base pairs. We assessed whether or not the known single-nt CoM was among the valid candidates which fulfilled the MFE-difference constraints for the given α/β values and how many such candidates are left. A low number implies a clear separation of the known CoM from other candidates.

**Figure 5:**
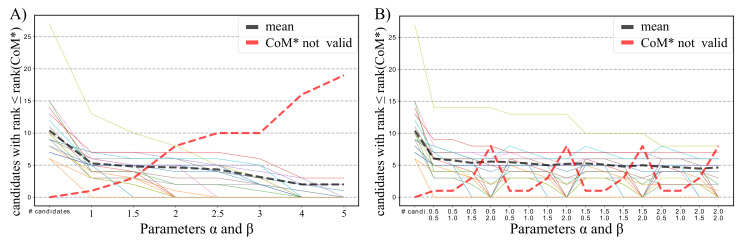
Effect of α/β thresholds on MFE-difference-based CoM classification. Each solid line represents the number of valid CoM candidates for an RNA pair with a rank not higher than the known CoM from literature (designated as CoM*; colors differentiate between the single CoMs). The left-most data points represent the overall numbers of CoM candidates in the RNA pair that harbors the respective CoM*. The black dotted line provides the average over all RNA pairs for α,β>0. The red dotted line depicts the number of RNA pairs for which the known CoM* does not fulfill the energy constraint. (**A**) Results for equal values of α and β; (**B**) Results for explicit value combinations of α and β in range [0.5, 2].

Results are depicted in Figure 5A. An α/β threshold of 1 prunes the candidate list already quite strongly. Increasing the thresholds results in a nearly linear loss of known CoMs among the remaining CoM candidates such that for value 2.5 already 10/31 known CoMs are considered invalid. On the other hand, no significant reduction of the average number of valid candidates can be observed for values between 1 and 2.5.

We thus performed another parameter screen to investigate non-uniform combinations of α and β, also summarized in Figure 5B. We observe that the effect of the energy-based candidate pruning is mainly governed by β, i.e., the energy difference to the mutant-only MFE. This is in accordance with the MFE difference statistics from Figure 4. As a result, we fix the default values of CopomuS’s E function to α=2 and β=1.

### 3.4. CopomuS Benchmark

Finally, we evaluated CopomuS’s ranking of known single-nt CoMs among its generated CoM candidates for different combinations of classification and sorting functions. We are interested in combinations for which many known CoMs fulfill all constraints with low (mean) ranking. Given our preliminary studies, we tested the effect of classification based on mfeCover and E (using α=2 and β=1) as well as sorting by minDeltaE. We restrict candidate generation to the flipping of GC and AU base pairs of the MFE RRI that are part of helices (no lonely base pairs or helix ends).

**Figure 6:**
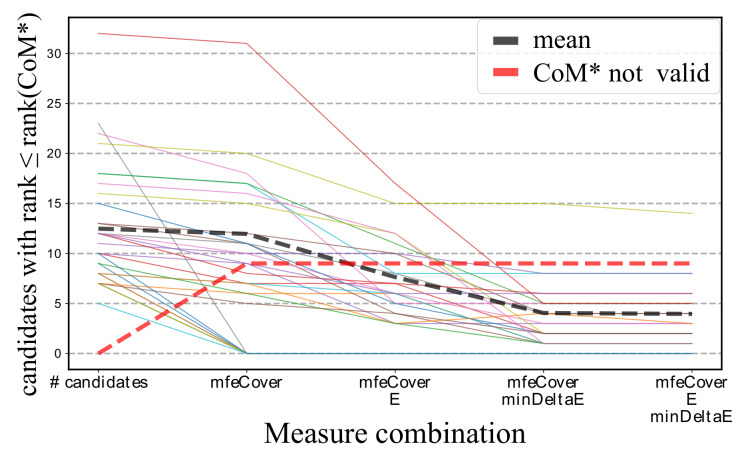
Effect of different classification and sorting combinations on CoM candidate set sizes. Each solid line represents the number of valid CoM candidates for an RNA pair with a rank not higher than the known CoM from literature (designated as CoM*; colors differentiate between the single CoMs). The left-most data points represent the overall numbers of CoM candidates. The black dotted line provides the average over all RNA pairs. The red dotted line depicts the number of RNA pairs for which the known CoM* does not fulfill the constraints.

Figure 6 summarizes the results. Classification based on mfeCover has only minor effects, while E-based pruning shows much stronger candidate set reductions without altering the number of known CoMs that are not fulfilling the constraints. minDeltaE sorting alone provides already very good results, which can be slightly enhanced when combined with E classification.

## 4. Discussion

### 4.1. Statistics of CoMs from Literature

The high abundance of GC mutations is related to the higher base pairing strength of GC base pairs compared to AU or GU base pairs. Thus, preventing the formation of a GC base pair via mutation will have on average a higher impact on RRI stability and thus interaction potential.

Within multi-nt CoMs, GC mutations are not as dominant as in single-nt CoMs. Here, the set of mutations of the multi-nt CoM often aims at the core of a long inter-molecular helix to prevent their formation in wildtype-mutant combinations. Thus, less focus on GC base pairs is possible and even GU base pairs are part of the CoM. If possible, no GU base pair is introduced in the mutant (only 1/86), since GU base pairs have the lowest stability contributions.

Flipping wildtype base pairs in mutants is an easy way to ensure base pair incompatibility in wildtype-mutant combinations and enables (on average) a similar stability of the wildtype-only and mutant-only RRI. Thus, we chose flipping as the standard mode of CoM candidate generation.

The high rate of CoMs found within the IntaRNA MFE predictions supports our choice to build CopomuS’s CoM selection based on IntaRNA RRI predictions. The prediction can fail e.g., if additional interaction partners, like RNA-binding proteins such as Hfq [14], are needed to guide or stabilize the RRI formation. In that case, IntaRNA’s prediction model has to be guided with respective constraints, which can be incorporated in the CopomuS workflow using its optional IntaRNA parameter file. Examples for such constraints are location constraints where RRIs are assumed, structure probing data from e.g., SHAPE experiments [15] or explicit seed interaction information. Another reason for missing a known CoM is that only one base pair pattern of an RRI is investigated, but sometimes slightly different patterns with equal energy within the same boundaries are possible. Since IntaRNA reports only one pattern per RRI boundaries, a known CoM present in an alternative pattern is missed.

The high abundance of stacked base pairs within the data set motivated the introduced CoM candidate filter capabilities of CopomuS, to exclude lonely and helix-end base pairs from the candidate generation.

### 4.2. Energy Profiling of CoMs from Literature

The pattern of average MFE differences of known CoMs (inversely) follows the desired relation of in vitro RRI signals depicted in Figure 1. That is, wildtype-mutant RRIs show on average lower stability (higher MFEs) compared to wildtype-only or mutant-only interactions. This, in concert with the less prominent pattern within the background model, strongly supports our hypothesis that we can indeed use IntaRNA MFE predictions to select highly potent CoM candidates. Furthermore, the higher mean minDeltaE values of CoMs motivate our final ranking of CoM candidates in CopomuS to identify mutations that show the desired pattern most prominently.

### 4.3. CopomuS Benchmark

Nine of all known CoMs from literature are not among the remaining valid candidates for all measures and combinations. Seven of these are considered invalid by mfeCover due to a lack of MFE RRI coverage (compare Figure 3B) and two are at helix ends and thus filtered during candidate generation.

As expected, the combination of mfeCover with the E classifier impacts the ranking less than combining mfeCover with minDeltaE; since the latter provides a high-resolution sorting instead. Nevertheless, a combination of all three functions provides the best average rank of about 4 and furthermore instantiates all rationales underlying CopomuS. Thus, we make the combination of mfeCover and E with a final sorting by minDeltaE the default ranking of CopomuS.

## 5. Conclusions

CopomuS implements an automated, objective evaluation strategy to identify compensatory mutations (CoMs) based on IntaRNA-based RRI stability analyses. That is, top-ranked CoMs show an in silico RRI stability pattern that follows the desired pattern of in vitro RRI potentials. The required scoring functions were derived from characteristics of CoMs used in successful RRI verification experiments known from literature. We could show that the introduced measures efficiently reduce the set of CoM candidates, while the known CoM was found on average within the top-5 candidates. That way, experimenters can easily and reproducibly pick promising candidates from the provided list without the need of time consuming manual RRI prediction and comparison. Therefore, we consider CopomuS a valuable tool to guide the difficult design of highly promising CoM-based RRI verification experiments.

Currently, CopomuS supports only single-nt CoM generation and evaluation. While potent multi-nt CoMs could be derived from top-ranked single-nt CoMs that can stack, no correct ranking of their combination can be done based on the current output. We therefore work on the extension of CopomuS to multi-nt CoM generation and testing, to also provide reliable selection criteria for this setup. Furthermore, we would like to evaluate CopomuS’s ranking order, which is currently not possible due to a lack of experimental data. That is, published CoM data is biased towards “valid” CoMs that were successfully used to verify an RRI. Non-successful CoMs or CoMs with low experimental effect are typically not published. Eventually, an extensive CoM feature and ranking evaluation would require in vitro measurements of multiple CoMs for the same RRI within the same experimental setup to be comparable.

## Abbreviations

The following abbreviations are used in this manuscript:

RRI	RNA-RNA interaction
CoM	compensatory mutation
MFE	minimum free energy
nt	nucleotide
ww, wm, mw, mm	sRNA-mRNA combinations of (w)ildtype and (m)utated variants
AUAU, AUCG, …	CoM classes where the first two letters encode the wildtype and the last two letters the mutated nts, e.g., AUCG encodes the mutation of either an AU or UA RRI base pair into either CG or GC

## Appendix A. Data

**Table A1:** Single-nucleotide CoMs from literature used for this study.

RNA-1	RNA-2	Mutation	Source
OxyS_NC_000913	b2731_NC_000913_-200+100	G102C&C-13G	[16]
CyaR_NC_000913	b2687_NC_000913_-200+100	A44U&U-7A	[17]
MicA_NC_000913	b0411_NC_000913_-200+100	C11G&G-46C	[18]
MicA_NC_000913	b0814_NC_000913_-200+100	C7G&G17C	[18]
RybB_NC_000913	b0805_NC_000913_-200+100	C2G&G-71C	[18]
RybB_NC_000913	b2594_NC_000913_-200+100	C2G&G4C	[18]
MicF_NC_000913	b0889_NC_000913_-200+100	C2G&G11C	[19]
SgrS_NC_000913	b1101_NC_000913_-200+100	G178C&C-19G	[20]
DsrA_NC_000913	b2741_NC_000913_-200+100	C16G&G-103C	[21]
RprA_NC_000913	b2741_NC_000913_-200+100	C42G&G-103C	[21]
ArcZ_NC_000913	b2741_NC_000913_-200+100	C70G&G-103C	[21]
ArcZ_NC_000913	b3546_NC_000913_-200+100	C69G&G-10C	[22]
CyaR_NC_000913	b1824_NC_000913_-200+100	G32C&C-11G	[3]
FnrS_NC_000913	b2531_NC_000913_-200+100	C47G&G-3C	[3]
RyhB_NC_000913	b3365_NC_000913_-200+100	C45G&G5C	[3]
RybB_NC_003197	STM1473_NC_003197_-200+100	C2G&G19C	[23]
MicF_NC_003197	STM0366_NC_003197_-200+100	C6G&G-31C	[24]
MicF_NC_003197	STM0959_NC_003197_-200+100	C6G&G7C	[24]
RybB_NC_003197	STM0413_NC_003197_-200+100	C2G&G-8C	[25]
RybB_NC_003197	STM0999_NC_003197_-200+100	C2G&G-39C	[25]
RybB_NC_003197	STM1070_NC_003197_-200+100	C2G&G31C	[25]
RybB_NC_003197	STM1572_NC_003197_-200+100	C2G&G25C	[25]
RybB_NC_003197	STM1732_NC_003197_-200+100	C2G&G19C	[25]
RybB_NC_003197	STM1995_NC_003197_-200+100	C2G&G19C	[25]
RybB_NC_003197	STM2267_NC_003197_-200+100	C2G&G-42C	[25]
RybB_NC_003197	STM2391_NC_003197_-200+100	C2G&G55C	[25]
CyaR_NC_003197	STM0833_NC_003197_-200+100	A43U&U-5A	[26]
SgrS_NC_003197	STM2945_NC_003197_-200+100	G176C&C5G	[27]
ArcZ_NC_003197	STM1682_NC_003197_-200+100	G70C&C22G	[28]
ArcZ_NC_003197	STM2970_NC_003197_-200+100	G70C&C-12G	[28]
MicC_NC_003197	STM1572_NC_003197_-200+100	C9G&G69C	[29]

**Table A2:** Multi-nucleotide CoMs from literature used for this study.

RNA-1	RNA-2	Mutation	Source
Spot42_NC_000913	b2702_NC_000913_-200+100	U23A&A-4U,C24G&G-5C,U25A&A-6U	[30]
Spot42_NC_000913	b3962_NC_000913_-200+100	G49C&C21G,U50A&A20U,A51U&U19A	[30]
Spot42_NC_000913	b4311_NC_000913_-200+100	G5A&C-20U,G6C&U-21G,U7G&A-22C	[30]
Spot42_NC_000913	b1302_NC_000913_-200+100	G55C&C-33G,G56A&C-34U,A57C&U-35G	[31]
Spot42_NC_000913	b2715_NC_000913_-200+100	G5A&C-25U,G6C&C-26G,U7G&A-27C	[31]
Spot42_NC_000913	b2801_NC_000913_-200+100	G49C&C-32G,U50A&A-33U,A51U&U-34A	[31]
Spot42_NC_000913	b3224_NC_000913_-200+100	G5A&C-54U,G6C&C-55G,U7G&A-56C	[31]
Spot42_NC_000913	b1901_NC_000913_-200+100	G5A&C-34U,G6C&C-35G,U7G&G-36U	[32]
MicA_NC_000913	b1130_NC_000913_-200+100	C7G&G7C,G8C&C6G,C9G&G5C,G10C&C4G	[33]
GcvB_NC_000913	b1130_NC_000913_-200+100	C158G&G-13C,U157A&A-14U,G156C&C-15G ,U155A&A-16U,C154G&G-17C	[34]
CyaR_NC_000913	b1740_NC_000913_-200+100	A40U&U-3A,G39A&C-2U	[17]
CyaR_NC_000913	b2666_NC_000913_-200+100	A40U&U7A,G39A&U8U,G38C&C9G	[17]
ArcZ_NC_000913	b1892_NC_000913_-200+100	U78A&G-60U,U77A&A-59U,G76G&C-58C,U75C&A-57G,G74C&U-56G,G73U&C-55A	[35]
OxyS_NC_000913	b1892_NC_000913_-200+100	A69U&U-18A,A68A&U-17U,U67C&A-16G ,A66C&U-15G,A65U&U-14A	[35]
RybB_NC_000913	b0721_NC_000913_-200+100	U12A&A-21U,C13G&G-22C	[36]
RyhB_NC_000913	b0721_NC_000913_-200+100	U51A&A-15U,A50U&U-14A	[36]
Spot42_NC_000913	b0721_NC_000913_-200+100	G13C&C-53G,G14C&C-54G	[36]
FnrS_NC_000913	b0755_NC_000913_-200+100	C47A&G-4U,U48A&A-5U,U49G&A-6C	[5]
FnrS_NC_000913	b1479_NC_000913_-200+100	U57A&A-13U,U58G&A-14C,U59A&A-15U	[5]
FnrS_NC_000913	b2153_NC_000913_-200+100	G5U&C-18A,G4C&C-19G	[5]
FnrS_NC_000913	b2303_NC_000913_-200+100	G5U&C-6A,G4C&C-5G	[5]
RyhB_NC_000913	b1656_NC_000913_-200+100	C49G&G-6C,C45G&G-3C,G44C&C-2G	[37]
RyhB_NC_000913	b0592_NC_000913_-200+100	G53U&C-7A,C54U&G-8A,U55C&A-9G	[38]
RyhB_NC_000913	b2155_NC_000913_-200+100	C47A&G-47U,A48U&U-48A,C49A&G-49U ,A50C&U-50G	[38]
Spot42_NC_000913	b1761_NC_000913_-200+100	C46G&G86C,C48G&G88C	[3]
RybB_NC_003197	STM0687_NC_003197_-200+100	C5G&G14C,A4U&U15A	[39]
MicA_NC_003197	STM4231_NC_003197_-200+100	A22C&U5G,A12G&U14C	[40]
GcvB_NC_003197	STM3930_NC_003197_-200+100	U84G&A-15C,G85A&C-16U,U86A&A-17U ,U87C&A-18G,U88A&A-19U	[41]

**Table A3:** sRNAs used within this study.

sRNA_Genome	Sequence
ArcZ_NC_000913	GTGCGGCCTGAAAAACAGTGCTGTGCCCTTGTAACTCATCATAATAATTTACGGCGCAGCCAAGATTTCCCTGGTGTTGGCGCAGTATTCGCGCACCCCGGTCTAGCCGGGGTCATTTTTT
ArcZ_NC_003197	GTGCGGCCTGAAAACAGGACTGCGCCTTTGACATCATCATAATAAGCACGGCGCAGCCACGATTTCCCTGGTGTTGGCGCAGTATTCGCGCACCCCGGTCAAACCGGGGTCATTTTTT
CyaR_NC_000913	GCTGAAAAACATAACCCATAAAATGCTAGCTGTACCAGGAACCACCTCCTTAGCCTGTGTAATCTCCCTTACACGGGCTTATTT
CyaR_NC_003197	GCTGAAAAACATAACCCATAAATGCTAGCTGTACCAGGAACCACCTCCTTGGCCTGCGTAATCTCCCTTACGCAGGCTTATTT
DsrA_NC_000913	AACACATCAGATTTCCTGGTGTAACGAATTTTTTAAGTGCTTCTTGCTTAAGCAAGTTTCATCCCGACCCCCTCAGGGTCGGGATTTTT
FnrS_NC_000913	GCAGGTGAATGCAACGTCAAGCGATGGGCGTTGCGCTCCATATTGTCTTACTTCCTTTTTTGAATTACTGCATAGCACAATTGATTCGTACGACGCCGACTTTGATGAGTCGGCTTTTTTTT
GcvB_NC_000913	ACTTCCTGAGCCGGAACGAAAAGTTTTATCGGAATGCGTGTTCTGGTGAACTTTTGGCTTACGGTTGTGATGTTGTGTTGTTGTGTTTGCAATTGGTCTGCGATTCAGACCATGGTAGCAAAGCTACCTTTTTTCACTTCCTGTACATTTACCCTGTCTGTCCATAGTGATTAATGTAGCACCGCCTAATTGCGGTGCTTT
GcvB_NC_003197	ACTTCCTGAGCCGGAACGAAAAGTTTTATCGGAATGCGTGTTCTGATGGGCTTTTGGCTTACGGTTGTGATGTTGTGTTGTTGTGTTTGCAATTGGTCTGCGATTCAGACCACGGTAGCGAGACTACCCTTTTTCACTTCCTGTACATTTACCCTGTCTGTCCATAGTGATTAATGTAGCACCGCCATATTGCGGTGCTTT
MicA_NC_000913	GAAAGACGCGCATTTGTTATCATCATCCCTGAATTCAGAGATGAAATTTTGGCCACTCACGAGTGGCCTTTT
MicA_NC_003197	GAAAGACGCGCATTTGTTATCATCATCCCTGTTTTCAGCGATGAAATTTTGGCCACTCCGTGAGTGGCCTTTT
MicC_NC_003197	GTTATATGCCTTTATTGTCACATATTCATTTTGTCGCTGGGCCATTGCGTTAACCTTTGCTTTCCAGCGTATAAATTGACAAGCCCGAACGGATGTTCGGGCTTTTTTT
MicF_NC_000913	GCTATCATCATTAACTTTATTTATTACCGTCATTCATTTCTGAATGTCTGTTTACCCCTATTTCAACCGGATGCCTCGCATTCGGTTTTTTTT
MicF_NC_003197	GCTATCATCATTAACTTTATTTATTACCGTCATTCACTTCTGAATGTCTGTTTACCCCTATTTCAACCGGATGCTTCGCATTCGGTTTTTTTT
OxyS_NC_000913	GAAACGGAGCGGCACCTCTTTTAACCCTTGAAGTCACTGCCCGTTTCGAGAGTTTCTCAACTCGAATAACTAAAGCCAACGTGAACTTTTGCGGATCTCCAGGATCCGC
RprA_NC_000913	ACGGTTATAAATCAACATATTGATTTATAAGCATGGAAATCCCCTGAGTGAAACAACGAATTGCTGTGTGTAGTCTTTGCCCATCTCCCACGATGGGCTTTTTTT
RybB_NC_000913	GCCACTGCTTTTCTTTGATGTCCCCATTTTGTGGAGCCCATCAACCCCGCCATTTCGGTTCAAGGTTGATGGGTTTTTT
RybB_NC_003197	GCCACTGCTTTTCTTTGATGTCCCCATTTTGTGGAGCCCATCAACCCCGCCATTTCGGTTCAAGGTTGGTGGGTTTTTT
RyhB_NC_000913	GCGATCAGGAAGACCCTCGCGGAGAACCTGAAAGCACGACATTGCTCACATTGCTTCCAGTATTACTTAGCCAGCCGGGTGCTGGCTTTT
SgrS_NC_000913	GATGAAGCAAGGGGGTGCCCCATGCGTCAGTTTTATCAGCACTATTTTACCGCGACAGCGAAGTTGTGCTGGTTGCGTTGGTTAAGCGTCCCACAACGATTAACCATGCTTGAAGGACTGATGCAGTGGGATGACCGCAATTCTGAAAGTTGACTTGCCTGCATCATGTGTGACTGAGTATTGGTGTAAAATCACCCGCCAGCAGATTATACCTGCTGGTTTTTTTT
SgrS_NC_003197	GATGAAGCAAGAGGAAGAGGTCACTATGCGCCAGTTCTGGTTGAGATATTTTGCCGCGACGGAAAAAACGTCCTGGCTGGCTTGCCTGAGCGCACCGCAGCGCTTAAAAATGCTCGCGGAACTGATGCAGTGGGAGGCGACCGATTGAAGCCAATTGCAGACATCATGTGTGACTGAGTATTGGTGTAGGCGATAGCCTAAAATCACCCGCCAGCAGATAATATCTGCTGGCTTTTTTT
Spot42_NC_000913	GTAGGGTACAGAGGTAAGATGTTCTATCTTTCAGACCTTTTACTTCACGTAATCGGATTTGGCTGAATATTTTAGCCGCCCCAGTCAGTAATGACTGGGGCGTTTTTTA

**Table A4:** genomic subsequences of NC_003197 -200+100 around start codon.

Locus_Genome_Range	Sequence	Gene
STM0366_NC_003197_-200+100	CGTTCATCTTATTAATAGTCAAACCAGATGATTGCGAGTGAGATCACAAAGCAGGGGCGTTTTAATCCGCGTTGTTACGCCGACAGAGCGGGGGCTGACTGGATTTTTCCAGTAATCTACACTACTTATTTAATCAGTCCGAACGGCCTTTTTGTTCTGATAAAGCGATGATGGCGTAATAATAAAACGAGGGTTTTGCTATGAAAACTGGCTACAAGGTTATGCTTGGCGCATTAGCGTTTGTCGTGACAAACGTTTATGCCGCAGAAATCATGAAAAAAACGGACTTTGATAAAGTCG	yahO
STM0413_NC_003197_-200+100	TCATGAAAGATAGTACTGTCGCCGCGTCTAAAATGCGCAAACGTGAACGCAATCGATTACGTAAATGATAGATATGTGAAACAAGACATATTTTTGTGAGCAATGATTTTTATAATAGGCTCCGCAGAAACACGAAATATTTAGAAACGCAAATTGCGTTCTTTTCACTCCCGCAAGGGATTTCAAACAGTGGCATACATATGAAAAAAACTTTACTCGCAGTCAGCGCAGCGCTGGCGCTCACCTCATCTTTTACTGCTAACGCAGCAGAAAATGATCAGCCGCAGTATTTGTCCGACT	tsx
STM0687_NC_003197_-200+100	AGCAGTCGAATGTAACAGAAAGCAATTAAATATGTGCGGTTGCTCATATTATTACATACTGGTTACAGAAAGAGATTGATAATTCGCATCGCGAAAAATAGTCTATTTAACGTAGTAAATGAGGTTTCTCAGCGCTACTTTTTATTTTTTCGCTGTTCGCTTTTGTCGGCAGCAATTTATACGTCAAAGAGGATTAACTTATGCGTACGTTTAGTGGCAAACGTAGTACGCTGGCTCTGGCTATCGCCGGTATCACAGCAATGTCGGGGTGGATCGTTGTTCCGCAGGCGCAAGCCTCCG	ybfM/chiP
STM0833_NC_003197_-200+100	CTGGATGAATGTATCGCGCCGCACGCGCATTATTGGTGCAATAAGCCGGAAAAGTGATGTTAATTGAATAAGATAGCGCGATATGGAAACGTTCTGTTACATGAAAGGCGCCCTTAGACACCGTGAATCGCAAAGAGTTTCCCATTAATTTTTGATATATTTAAAACTTAGGACTTACTTGAAGCACATTTGAGGTGGTTATGAAAAAAATTGCATGTCTTTCAGCACTGGCCGCTGTTCTGGCTTTTTCCGCAGGTACTGCAGTAGCTGCTACTTCTACCGTTACCGGTGGTTACGCTC	ompX
STM0959_NC_003197_-200+100	TGAAATCTACGCATGGCGTGGACAGACGCCATTCGTGATGTCGATAGCTGCCGCGAGGCAACGGTCTTCTCACCATAGACCAGGCATTGCGCGCCGTTAATCCCTCTGGGTTTCGGTCTATCGTGATGGGCAGCGACTCTGAACAGTGATGTGAGTAGAGTCAGGCAGGAGTAGGGAAGGAATACAGAGAGACAATAATAATGGTAGATAGCAAGAAGCGCCCTGGCAAAGATCTCGACCGTATCGATCGTAACATTCTTAATGAACTGCAAAAGGATGGGCGTATTTCCAACGTCGAGC	lrp
STM0999_NC_003197_-200+100	ACATATTATTTCCTTTTGAAACCAAATCTTTATCTTTGTAGCACTTTCACGGTAGCGAAACGTTAGTTTGAATGGAAAGATGCCTGTCAGACACATAAAGACACCAAACTCTCATCAATATTTCTGTAAAGTTTTATTGACGGAATTTATTGACGGCAGTGGCAGGTGTCATATAAAAAAACCAATGAGGGTAATAAATAATGATGAAGCGCAAAATCCTGGCAGCGGTGATCCCTGCCCTGCTGGCTGCTGCAACCGCAAACGCAGCAGAAATTTATAATAAAGATGGTAATAAGCTGG	ompF
STM1070_NC_003197_-200+100	TGTTTTTTTCACATGTCTGACGGAGTTCACACTTGTAAGTTTCCAACTACGTTGTAGACTTTACATCGCCAGGGGTGCTCAGCATAAGCCGTAGATATCGGTAGAGTAACTATTGAGCAGATCCCCCGGTGAAGGATTTAACCGTGTTATCTCGTTGGAGATATTCATGGCGTATTTTGGATGATAACGAGGCGCAAAAAATGAAAAAGACAGCTATCGCGATTGCAGTGGCACTGGCTGGTTTCGCTACCGTAGCGCAGGCCGCTCCGAAAGATAACACCTGGTACGCTGGTGCTAAAC	ompA
STM1473_NC_003197_-200+100	CACATACATGAATACATAATAACAAATATATTCACCATAAATATATGCGTTTCCGATAGTAACTTTTGTATTAATTAATAACATATAAGAAAAGTTAGCATTTGCTGAAATAATATTATTCAGATTAGGATGCCTTTGATTCAACGAATCTGTAGAAGTTCAATCTTTTGCAAATAAGTTAAGTTTTTAAGGATAAAAAAATGAAAAGAAAAGTATTGGCACTTGTCATCCCGGCTCTGCTGGCTGCTGGCGCAGCACACGCCGCTGAAATTTATAACAAAGACGGCAACAAACTGGACC	ompN
STM1572_NC_003197_-200+100	TTAGACAGTCCCTATTTGAATTAATACTCTCAAATGTATTAAGGAGATCTCGATCACACAAATTAAAATAATTTGTAATCTTATGAAACTTATTATTGAACTTATGCCACTCCGTCATTTAAAAATAGTCTGCCATTGACAAACGCCTCGTTTAACAATGGTTGAGGAAACACGCTAAGAAAATTATAAGGATTATTAAAATGAAACTTAAGTTAGTGGCAGTGGCAGTGACTTCCCTGTTGGCAGCAGGCGTTGTAAATGCAGCCGAGGTATATAACAAAGACGGCAATAAACTGGATC	ompD
STM1682_NC_003197_-200+100	CATGCGCTTCGCTTCGGCTACCACGCGCAATAACAAAAGGCGTATGTAACGGCCAGGTTTCCTCATATACCTTAACAGATCTCATCTCTTCCCCTCTGATAGCGCCGGACGCGGCTTGCTACAAATAGTATTTGCCGTGTTAATGAATAATGACTTAAACTGGATTTCGACGTTAACTATAAGTAAATAGGAACATAATTATGTCACAGACTGTACATTTCCAGGGTAACCCGGTCACCGTTGCCAACGTTATTCCGCAGGCTGGTAGCAAAGCACAGGCTTTTACTCTTGTCGCAAAAG	tpx
STM1732_NC_003197_-200+100	GAGCAGACAAATATTTGCATAGCGTGAATATGTCAAAATTGATCTGAATTCCTATAACCAGGATTTTCAATACAAGTTCTAAATTAATCTGGATCAATAAATGTTAAATTATAAGAACAAATGTGATCTGTATTAGATCACTTATTACTTCATTGTGGGTATATTCATCACGCTTTTATAACCATAACGATGGAGCGGGTATGAAAAAATTTACAGTGGCGGCACTGGCGTTAACAACTCTTCTCTCAGGCAGCGCGTTCGCGCACGAAGCCGGAGAATTCTTTATGCGTGCAGGTCCGG	ompW
STM1995_NC_003197_-200+100	GATAATTATAGAATATATATTCTTAGTTACTTATATAGTCTGTATTATAAAAAACCAAACAGAAACAAATTGAAATATTTTAAATACCTTTGTTACATGTTATTTTTTAAATTCCATGAACTTCATAGAATAGTATCAATTTGTAGTTTTGTTGAAGTGGCTACATATTCATATAAATTATTATCATAAGGGAATACATAATGAACAGAAAAGTTCTGGCACTGCTTGTCCCGGCGTTATTAGTGGCAGGCGCAGCAAATGCGGCTGAAGTTTATAATAAAAATGGCAACAAACTCGACC	ompS
STM2267_NC_003197_-200+100	GCTTTAAAAAAGTTCCGTAAAATTCATATTTTGAAACATCTATGTAGATAACTGTAACATCTTAAAAGTTTTAGTATCATATTCGTGTTGGATTATTCTGTATTTTTGCGGAGAATGGACTTGCCGACTGGTTAATGAGGGTTAACCAGTAAGCAGTGGCATAAAAAAGCAATAAAGGCATATAACAGAGGGTTAATAACATGAAAGTTAAAGTACTGTCCCTCCTGGTACCAGCTCTGCTGGTGGCGGGCGCAGCGAATGCGGCTGAAATTTATAATAAAGACGGCAACAAATTAGACC	ompC
STM2391_NC_003197_-200+100	TTAAGCCCGCCGATTTTGCCAGCCAGATCTCGTTTCTAAGATCACAATTGAAAAAACTTATAAACATACTTGCAACATTCTAGCTGGTCAGACCTATACTCTCGCCACTGGTCTGATTTCTAAGTCGTACCGCAGACCCTACACTTCGCGCTCCTGTTACAGCATGTAACATAGTTTGTATAAAAATAATCAATGAGGTTATGGTCATGAGCCAGAAAACCCTGTTTACAAAGTCTGCTCTCGCAGTCGCAGTGGCAATCATCTCCACCCAGGCCTGGTCTGCAGGCTTTCAGTTAAACG	fadL
STM2945_NC_003197_-200+100	AACGACCATTTGCGGCGAATCATCTACCTTTTGTCTGAATTATCGTCACCACAAAGGATTACCAACCATAAATGTGCTGTATTAATAATGTCGTTCAAATTCTCTCCTGTAGTAAACTTTATCTGTTTAATAAAAAAGAGAGAATTGAACGATATATTTTACTCCGGATATTGAATAATATAAATTTGAAGGAAAATATTATGCCAGTCACTTTAAGCTTCGGTAATCATCAAAATTATACGCTTAATGAAAGTCGGCTTGCTCATCTGTTAAGCGCAGATAAAGAAAAAGCAATCCATA	sopD
STM2970_NC_003197_-200+100	TCCATGTAAGAAGCGGATTATTGCATTTGAGATCGGGATCACTGATAGATTCATCACTTAAATGTATCTTTCCGCCCGAAAATTATTACGGCGAAAAATTATATAAAAAGCGTCCCTAAGCAGATTTCATTTTACGATCAGGTCTTTTTTCATTGGATTAGACCAGCAACCTGATTTTTAGCATCCTCCAGGAGAAATAGATGGAAACCACTCAGACCAGCACTATTGCTTCGATTGACTCTCGAAGCGCATGGCGCAAAACGGATACCATGTGGATGCTGGGCCTTTACGGCACGGCTA	sdaC
STM3930_NC_003197_-200+100	CTTCTGATGACTTGAGCAGCGGATTGTGCTTATGGTGCTGCTCATTTACAACATAATCGATGATTTCTTACACAATAAGTGCATTTTTTTAATGCTCCATTTGCCATTTGTCCAAATTTAAGAAAATATTCGCAACAATCGATGTACCCATAACAATAACCGGTACTACCGGAACCGTTGCAAACACGACATGAGGATTTATGGCAGAGAAAAAACCGGAGCTACAGCGTGGGCTGGAAGCTCGTCATATTGAATTGATTGCCCTCGGGGGCACCATCGGCGTCGGACTCTTTATGGGCG	yifK
STM4231_NC_003197_-200+100	GTTGGTAGAAGAGGGCGCCACATTCGCTATCGGCCTGCCGCCAGAGCGCTGTCATCTGTTCCGCGAGGATGGCAGCGCATGTCGTCGTCTGCATCAAGAGCCGGGTGTTTAAGGCCTCCATAAAAAAACGAAACGCAAAACCATTCGCAGTTTTAGAAGGTGGCAGCGTTTAAAGAAAAGCAATGATCTCAGGAGATAGAATGATGATTACTCTGCGCAAACTCCCACTGGCGGTTGCTGTCGCAGCGGGCGTAATGTCCGCTCAGGCAATGGCTGTCGATTTCCACGGTTACGCCCGTT	lamB

**Table A5:** genomic subsequences of NC_000913 -200+100 around start codon (continued in Table A6).

Locus_Genome_Range	Sequence	Gene
b0411_NC_000913_-200+100	AGGGCGAAAGTCAGTACAATCCCCGCCCGAATGTGTGTAAACGTGAACGCAATCGATTACGTAAATGATAGAACTGTGAAACGAAACATATTTTTGTGAGCAATGATTTTTATAATAGGCTCCTCTGTATACGAAATATTTAGAAACGCAATTTGCGCCTTTTTCACTCCCGCAAGGGATTTTCAAACAGTGGCATACATATGAAAAAAACATTACTGGCAGCCGGTGCGGTACTGGCGCTCTCTTCGTCTTTTACTGTCAACGCAGCTGAAAACGACAAACCGCAGTATCTTTCCGACT	tsx
b0592_NC_000913_-200+100	ATAAGCGCAATGTGATGTCCTGCGCCGTTCTGCCCCCTCTCCCTTCCAGGGTGAGGGCTGGGGTGAGGGTTAATGTTCGCACCAGTGCTGGCTGTTCCCCTCACCCTAACCCTCTCCCCAAAGGGGCGAGGGGACGGATTGTGCGCTTTGTCGAATTTGTCATTACGCCCTTAACCTTATTAATAACAGGAAGCTGATTTGTGAGACTCGCCCCGCTCTACCGCAACGCCCTTCTATTAACAGGACTTTTGCTTTCAGGAATAGCCGCAGTTCAGGCCGCTGACTGGCCGCGTCAGATTA	fepB
b0721_NC_000913_-200+100	TCCCGAGCCACCCAGCGTTGTAACGTGTCGTTTTCGCATCTGGAAGCAGTGTTTTGCATGACGCGCAGTTATAGAAAGGACGCTGTCTGACCCGCAAGCAGACCGGAGGAAGGAAATCCCGACGTCTCCAGGTAACAGAAAGTTAACCTCTGTGCCCGTAGTCCCCAGGGAATAATAAGAACAGCATGTGGGCGTTATTCATGATAAGAAATGTGAAAAAACAAAGACCTGTTAATCTGGACCTACAGACCATCCGGTTCCCCATCACGGCGATAGCGTCCATTCTCCATCGCGTTTCCG	sdhC
b0755_NC_000913_-200+100	GTTACGCCCTCGTCATGAGGGCTTTATCTCATATTGTTCAAATCACCAGCAAACACCGACATATTTGCAACTCAATATTCACAACAACCTTACACTGCGCCACTATTTTCGCTATGGTTATGCGTAAGCATTGCTGTTGCTTCGTCGCGGCAATATAATGAGAATTATTATCATTAAAAGATGATTTGAGGAGTAAGTATATGGCTGTAACTAAGCTGGTTCTGGTTCGTCATGGCGAAAGTCAGTGGAACAAAGAAAACCGTTTCACCGGTTGGTACGACGTGGATCTGTCTGAGAAAG	gpmA
b0805_NC_000913_-200+100	TCAAATATGAACTCAATGTAAATAAATGTATTTCTTTTTCGCGCAATGGGTGATAGAAAATCGCTCCAAGTGATAATGCTTATCAAAATTATTATCACTTTCACGAGCACTATCACGGGATTAACAGTGGCATCGCATCCGCAGAGAGGCTTTCTCGTGGCAGTGAAAATTTCAACATATAAGAAAAAGTCACCTGCAAAATGGAAAACAATCGCAATTTCCCTGCCAGACAATTTCATTCGCTCACGTTCTTTGCCGGTCTTTGTATTGGCATCACGCCTGTGGCTCAGGCACTCGCCG	fiu
b0814_NC_000913_-200+100	CTGGATGAATGACAGGGAAAACATGCGTAATACTTACGCAGTTCTCTGAAAAAGTGATTTAAATTTAGATGGATAGCGGTGTATGGAAACGTTCTGTTACATGAAATGGCCCGTTAGACATCACAAATCGCGAAGAGTTTCCCATTAATTTTTGATATATTTAAAACTTAGGACTTATTTGAATCACATTTGAGGTGGTTATGAAAAAAATTGCATGTCTTTCAGCACTGGCCGCAGTTCTGGCTTTCACCGCAGGTACTTCCGTAGCTGCGACTTCTACTGTAACTGGCGGTTACGCAC	ompX
b0889_NC_000913_-200+100	GTGAAATCTACGTATGGCGTGGACAGACGCCATTCGTGATGTCGATAGCTGCCACAAGGCAACGGTCTTCTCACCGTAGACCCAGGCATTGCGCGCCGTGAATCTTCATGATTTCGGTCTATCGTGACGGGTAGCGACTCTGAACAGTGATGTTTCAGGGTCAGACAGGAGTAGGGAAGGAATACAGAGAGACAATAATAATGGTAGATAGCAAGAAGCGCCCTGGCAAAGATCTCGACCGTATCGATCGTAACATTCTTAATGAGTTGCAAAAGGATGGGCGTATTTCTAACGTCGAGC	lrp
b1101_NC_000913_-200+100	CATATGTTTTGTCAAAATGTGCAACTTCTCCAATGATCTGAAGTTGAAACGTGATAGCCGTCAAACAAATTGGCACTGAATTATTTTACTCTGTGTAATAAATAAAGGGCGCTTAGATGCCCTGTACACGGCGAGGCTCTCCCCCCTTGCCACGCGTGAGAACGTAAAAAAAGCACCCATACTCAGGAGCACTCTCAATTATGTTTAAGAATGCATTTGCTAACCTGCAAAAGGTCGGTAAATCGCTGATGCTGCCGGTATCCGTACTGCCTATCGCAGGTATTCTGCTGGGCGTCGGTT	ptsG
b1130_NC_000913_-200+100	GAGCTATCACGATGGTTGATGAGCTGAAATAAACCTCGTATCAGTGCCGGATGGCGATGCTGTCCGGCCTGCTTATTAAGATTATCCGCTTTTTATTTTTTCACTTTACCTCCCCTCCCCGCTGGTTTATTTAATGTTTACCCCCATAACCACATAATCGCGTTACACTATTTTAATAATTAAGACAGGGAGAAATAAAAATGCGCGTACTGGTTGTTGAAGACAATGCGTTGTTACGTCACCACCTTAAAGTTCAGATTCAGGATGCTGGTCATCAGGTCGATGACGCAGAAGATGCCA	phoP
b1302_NC_000913_-200+100	AGCCGGACGTTTGATTGCCGAACTGCTGCGCGGCGACGCCGAACGTTTCGATGCCTTCGCCAATCTGCCGCATTACCCGTTCCCCGGCGGGCGCACGCTGCGTGTGCCGTTTACCGCGATGGGCGCGGCGTATTACAGCCTGCGCGATCGTCTGGGCGTTTAATTTCCGATTAACCGTGAAGAGTCAAAAGGTGTGAAACATGAGCAACAATGAATTCCATCAGCGTCGTCTTTCTGCCACTCCGCGCGGGGTTGGCGTGATGTGTAACTTCTTCGCCCAGTCGGCTGAAAACGCCACGC	puuE
b1479_NC_000913_-200+100	TAGTAAATAACCCAACCGGCAGAAAACGCCCCGCTGAAAAGTAATTCATAACCATCAGTCCTCAATGACGATTAAACACCATTGCCTGCGCAATGGTGTTTTTGTTTTTATCTGCTTTATACTTGAGGCCGACGCCCTGGCGGTAAAGCAAAGACGATAAAAGCCCCCCAGGGATGGATATTCAAAAAAGAGTGAGTGACATGGAACCAAAAACAAAAAAACAGCGTTCGCTTTATATCCCTTACGCTGGCCCTGTACTGCTGGAATTTCCGTTGTTGAATAAAGGCAGTGCCTTCAGCA	maeA
b1656_NC_000913_-200+100	TCTCAGTGAAGACTACTGGCAGCGCCACTATGTTGGCGCTCGTCGGGTAATGACCCCAAAAACACTTCGCTAAAACTTTACCCTGTTGTTACGGCAACAGGGTAAGTTCATCTTTTGTCTCACCTTTTAATTTGCTACCCTATCCATACGCACAATAAGGCTATTGTACGTATGCAAATTAATAATAAAGGAGAGTAGCAATGTCATTCGAATTACCTGCACTACCATATGCTAAAGATGCTCTGGCACCGCACATTTCTGCGGAAACCATCGAGTATCACTACGGCAAGCACCATCAGA	sodB
b1740_NC_000913_-200+100	TTCCGTCCTCTTGTTTATCAGCGTGTTAGATAAGCCTGGAATACATTGGGCGCTTTTTCAAGCCCGTGAACGAAACGGCTCCGCTTTCAGAGGATTCCTGTATGACGTTTTAACCACCATTCAGCCCGCTGTCGCTTGTCGTTTCAGTAGCAACGGGTTAGCTTTAAGGAAGTTTTGTCTTTTCTGTCTGGAGGGGTTCAATGACATTGCAACAACAAATAATAAAGGCGCTGGGCGCAAAACCGCAGATTAATGCTGAAGAGGAAATTCGTCGTAGTGTCGATTTTCTGAAAAGCTACC	nadE
b1761_NC_000913_-200+100	TAACGGTAGCCGGGTGGCAAAACTTTAGCGTCTGAGTTATCGCATTTGGTTATGAGATTACTCTCGTTATTAATTTGCTTTCCTGGGTCATTTTTTTCTTGCTTACCGTCACATTCTTGATGGTATAGTCGAAAACTGCAAAAGCACATGACATAAACAACATAAGCACAATCGTATTAATATATAAGGGTTTTATATCTATGGATCAGACATATTCTCTGGAGTCATTCCTCAACCATGTCCAAAAGCGCGACCCGAATCAAACCGAGTTCGCGCAAGCCGTTCGTGAAGTAATGACCA	gdhA
b1824_NC_000913_-200+100	GCCAGTTTAAGTATCTGCCTGAACTGGCAAGGTTAAGCACAATGATATATCGGCGCGTATTCCGTTGCATAAGTGTGCAAAAAAAGTGGAAGACGTATCGAGATTTGTGCGTCTGATCGAGACATGTTTAAAAATGGCTTGCCATAATTAACGTTGTATGTGATAACAGATTTCGGGTTAAACGAGGTACAGTTCTGTTTATGTGTGGCATTTTCAGTAAAGAAGTCCTGAGTAAACACGTTGACGTTGAATACCGCTTCTCTGCCGAGCCTTATATTGGTGCCTCATGCAGTAATGTGT	yobF
b1892_NC_000913_-200+100	TCGATTTAGGAAAAATCTTAGATAAGTGTAAAGACCCATTTCTATTTGTAAGGACATATTAAACCAAAAAGGTGGTTCTGCTTATTGCAGCTTATCGCAACTATTCTAATGCTAATTATTTTTTACCGGGGCTTCCCGGCGACATCACGGGGTGCGGTGAAACCGCATAAAAATAAAGTTGGTTATTCTGGGTGGGAATAATGCATACCTCCGAGTTGCTGAAACACATTTATGACATCAACTTGTCATATTTACTACTTGCACAGCGTTTGATTGTTCAGGACAAAGCGTCCGCTATGT	flhD
b1901_NC_000913_-200+100	TCCCGCTAAATTTATGCACGTTCTCACTGTAATTCTGCGATGTGATATTGCTCTCCTATGGAGAATTAATTTCTCGCTAAAACTATGTCAACACAGTCACTTATCTTTTAGTTAAAAGGTAATGCTTTGTTTTCCGATTAATTTAACGAATGTCATTCGTTTTTGCCCTACACAAAACGACACTAAAGCTGGAGAGAACCATGCACAAATTTACTAAAGCCCTGGCAGCCATTGGTCTGGCAGCCGTTATGTCACAATCCGCTATGGCGGAGAACCTGAAGCTCGGTTTTCTGGTGAAGC	araF
b2153_NC_000913_-200+100	CTGTGAGTAACTTTCACTTCCGTATTTGCATAACGATGTTTTAACATCTGCTGATGAAAGGCAGCGGCAATTACAATAATTATCGCTGTGAATACTGGATTATGTGCGCCGCCTCACGCACAATAATCAGGCTGTAAATCAGCTTAATAACTTTGCCCCCACGCAGGGCGGAGGCGTCACACCTGCAGGAGAAATCATAAATGCCATCACTCAGTAAAGAAGCGGCCCTGGTTCATGAAGCGTTAGTTGCGCGAGGACTGGAAACACCGCTGCGCCCGCCCGTGCATGAAATGGATAACG	folE
b2155_NC_000913_-200+100	GGATTGATAATTGTTATCGTTTGCATTATCGTTACGCCGCAATCAAAAAAGGCTGACAAATCAGAGGCTGTTCCGGCTTTCTGGGATGATCACCTGCATAAAAAATAAGTCCACCGCGATGCTGCCGTACGCAAGGGGACGTGAAGAAGATGTGAGCGATAACCCATTTTATTTTCGTAGTTACCTCATGGAGATATGGAATGTTTAGGTTGAACCCTTTCGTACGGGTCGGGCTGTGTTTGTCCGCTATTTCTTGTGCATGGCCTGTGTTAGCGGTCGATGATGATGGCGAAACGATGG	cirA
b2303_NC_000913_-200+100	TGGGTTAATGCCTGGACTCGCCAGCGAATTGACCTAGCAATGTATCCGGCAGTCAAGAACTGGCATGAGCGGATCCGTTCGCGCCCTGCCACCGGGCAGGCACTGCTAAAAGCACAACTCGGTGATGAGCGTTCGGATAGTTAACAGAAACAGGTTCTCGTGTATTATTTCATCCTAAGTAAAACAACGGAGAACCTGCAATGGCACAACCTGCCGCTATTATTCGTATAAAGAACCTTCGTTTGCGTACGTTTATCGGAATTAAGGAAGAAGAAATTAACAACCGTCAGGATATTGTTA	folX
b2531_NC_000913_-200+100	ATGGCGTTCACGCCGCATCCGACAACAGGTACAAACGCCACGATAAAAAAATGGCACTGAAGGTTAAATACCCGACTAAATCAGTCAAGTAAATAGTTGACCAATTTACTCGGGAATGTCAGACTTGACCCTGCTATGCAATACCCCCACTTTTACAATAAAAAACCCCGGGCAGGGGCGAGTTTGAGGTGAAGTAAGACATGAGACTGACATCTAAAGGGCGCTATGCCGTGACCGCAATGCTTGACGTTGCGCTCAACTCTGAAGCGGGCCCGGTACCGTTGGCTGATATTTCCGAAC	iscR
b2594_NC_000913_-200+100	CCCCGAGCAACCCGCCAAAAACAGGCTTAGTGTGGCGGCTGCCACCAGATATTTCATGCGCGTCATGACGTTTTGACTTTCCTCAAAATGTAATACGGGAGATTCTCTGTTCCTGCTCCCGGTTAAGACCAGCTACAATAGCACACTATATTAAACGGCAAAGCCGTAAAACCCCAACGATAAACGAAGAAGCAGTATATATGGCACAACGAGTACAGCTCACTGCAACGGTGTCCGAAAACCAACTCGGTCAACGCTTAGATCAGGCTTTGGCCGAAATGTTCCCGGATTATTCACGTT	rluD
b2666_NC_000913_-200+100	TTGCGCGAGTTCAGTCATATTTATTTAAGTATTTTCTAAATTAAGTAAACTCTAAACTAAAAATGCAACATATACCAGCCTCAGCAGCGTAAATGAGAGTAAAAGCGTAAGCTGAAACTGGCAGGCTCCGCTAAAATTACTACGCTTAAGAGATAAAATCTCTTTTTAAACAATGAGTAATTTTCTTATAGGGAGTACATATGGGTTTCTGGAGAATCGTCATCACCATCATTCTGCCGCCGCTCGGCGTGCTGCTCGGTAAAGGGTTCGGTTGGGCGTTCATTATTAATATTCTGTTGA	yqaE
b2687_NC_000913_-200+100	GAAGCCGCTGATACCGAACCGTTTGCGGTGTGGCTGGAAAAACACGCCTGACAGAAAAGAAAAAGGCCACTCGTGAGTGGCCAAAATTTCATCTCTGAATTCAGGGATGATGATAACAAATGCGCGTCTTTCATATACTCAGACTCGCCTGGGAAGAAAGAGTTCAGAAAATTTTTAAAAAAATTACCGGAGGTGGCTAAATGCCGTTGTTAGATAGCTTCACAGTCGATCATACCCGGATGGAAGCGCCTGCAGTTCGGGTGGCGAAAACAATGAACACCCCGCATGGCGACGCAATCA	luxS

**Table A6:** genomic subsequences of NC_000913 -200+100 around start codon (continuation of Table A5).

Locus_Genome_Range	Sequence	Gene
b2702_NC_000913_-200+100	CGCACAAGGAAGCGGTAGTCACTGCCCGATACGGACTTTACATAACTCAACTCATTCCCCTCGCTATCCTTTTATTCAAACTTTCAAATTAAAATATTTATCTTTCATTTTGCGATCAAAATAACACTTTTAAATCTTTCAATCTGATTAGATTAGGTTGCCGTTTGGTAATAAAACAATAAATCCTGAAGGAGAGAACAATGATAGAAACCATTACTCATGGTGCAGAGTGGTTTATCGGGCTGTTCCAAAAGGGCGGAGAGGTGTTTACCGGGATGGTGACCGGCATTCTTCCGCTGT	srlA
b2715_NC_000913_-200+100	TGCACAATCGGCGGGAAAAATATTCAGGTGACCGGTTTCACAAATATAAAAAATGAACAATTCACTCTCTTGCTTATTTAGTGACAACTATTCATGATTTTGTGAAACCGGTTTCTTAATTCCGTTTCAGCATCGGCATTTTTCCGTCACGTCGACTGATAACAACTACATCTACCCTACTGATAACAGGATAAAATCCGATGGCCAAAAATTATGCGGCGCTGGCACGCTCGGTGATAGCGGCACTGGGCGGCGTTGATAACATCTCGGCGGTCACGCACTGTATGACGCGGTTGCGCT	ascF
b2731_NC_000913_-200+100	ACTGGGGAAAGACGCGGCGCTGATTGGTGAAGTGGTGGAACGTAAAGGTGTTCGTCTTGCCGGTCTGTATGGCGTGAAACGAACCCTCGATTTACCACACGCCGAACCGCTTCCGCGTATATGCTAATAAAATTCTAAATCTCCTATAGTTAGTCAATGACCTTTTGCACCGCTTTGCGGTGCTTTCCTGGAAGAACAAAATGTCATATACACCGATGAGTGATCTCGGACAACAAGGGTTGTTCGACATCACTCGGACACTATTGCAGCAGCCCGATCTGGCCTCGCTGTGTGAGGCTC	fhlA
b2741_NC_000913_-200+100	CGGGAACAACAAGAAGTTAAGGCGGGGCAAAAAATAGCGACCATGGGTAGCACCGGAACCAGTTCAACACGCTTGCATTTTGAAATTCGTTACAAGGGGAAATCCGTAAACCCGCTGCGTTATTTGCCGCAGCGATAAATCGGCGGAACCAGGCTTTTGCTTGAATGTTCCGTCAAGGGATCACGGGTAGGAGCCACCTTATGAGTCAGAATACGCTGAAAGTTCATGATTTAAATGAAGATGCGGAATTTGATGAGAACGGAGTTGAGGTTTTTGACGAAAAGGCCTTAGTAGAACAGG	rpoS
b2801_NC_000913_-200+100	ATGGTAGTCACATAAAGTCACCTTCTAGCTAATAAGTGTGACCGCCGTCATATTACAGAGCGTTTTTTATTTGAAAATGAATCCATGAGTTCATTTCAGACAGGCAAATATTCACTGATATGAAGCCCGAACTCGCTGGTTTTGCACTTTTGAAAACATAACCGATTACGTGCTTAAGCTTCTGAACCTAAGAGGATGCTATGGGAAACACATCAATACAAACGCAGAGTTACCGTGCGGTAGATAAAGATGCAGGGCAAAGCAGAAGTTACATTATTCCATTCGCGCTGCTGTGCTCAC	fucP
b3224_NC_000913_-200+100	CGCTGTGCCGCAAACCGTTTGGACCGGTAGATGAAAAATATCTGCCAGAACTGAAGGCGCTGGCCCAGCAGTTGATGCAAGAGCGCGGGTGAGTTGTTTCCCCTCGCTCGCCCCTACCGGGTGAGGGGAAATAAACGCATCTGTACCCTACAATTTTCATACCAAAGCGTGTGGGCATCGCCCACCGCGGGAGACTCACAATGAGTACTACAACCCAGAATATCCCGTGGTATCGCCATCTCAACCGTGCACAATGGCGCGCATTTTCCGCTGCCTGGTTGGGATATCTGCTTGACGGTT	nanT
b3365_NC_000913_-200+100	ATCTATTTCTATAAACCCGCTCATTTTGTCTATTTTTTGCACAAACATGAAATATCAGACAATTCCGTGACTTAAGAAAATTTATACAAATCAGCAATATACCCATTAAGGAGTATATAAAGGTGAATTTGATTTACATCAATAAGCGGGGTTGCTGAATCGTTAAGGTAGGCGGTAATAGAAAAGAAATCGAGGCAAAAATGAGCAAAGTCAGACTCGCAATTATCGGTAACGGTATGGTCGGCCATCGCTTTATCGAAGATCTTCTTGATAAATCTGATGCGGCCAACTTTGATATTA	nirB
b3546_NC_000913_-200+100	CTAAAGTCTCTTTTCAAACTTGCATTTTTGTAAATTTGTGCTTCATGCACACTCTTTCCCCACACTTTTTCCCTTTGCTGTGGTCTACTTATTCGCGCGTGTAGATTTTACTTATCTGACTACCTCCGCACTTTTTCCCTGCCGGGCCTGAAAAGCCACTAAGCAGGGTGTTATCACCTGTTTGTCCAGGGTTTGTTTGCATGAGATACATCAAATCGATTACACAGCAGAAGCTGAGCTTTTTGCTTGCAATCTATATTGGCCTTTTTATGAATGGCGCGGTTTTTTACCGCCGCTTCG	eptB
b3962_NC_000913_-200+100	TACGTACAGCGGAAACCTGCCGCTTAAACGGAGAGTATCGTCGATAAAAATCCAATAAAACGTCAGGGCAAAAGTAAGAAACAGACAAAGCAAAGGCCGCTCAGGATATAGCCAGATAAATGACGGGGATCAATTGGCTTACCCGCGATAAAATGTTACCATTCTGTTGCTTTTATGTATAAGAACAGGTAAGCCCTACCATGCCACATTCCTACGATTACGATGCCATAGTAATAGGTTCCGGCCCCGGCGGCGAAGGCGCTGCAATGGGCCTGGTTAAGCAAGGTGCGCGCGTCGCAG	sthA
b4311_NC_000913_-200+100	GTATTTAATCTGGATCTCTGTTTATTTAAATAATGTGAAAAGAGATTTTTCACAGGAGACCTTATACAAAAAAATATAAAATACAGCTACCGGTTGCCAAAGACACTATAAGCCTGGCAAAAAAATATTACACAACATAAATGCTAATTGTTTATGCGGGCTTTGTATTGCTTTCTGTATCCTACAAATGAGTGAAATTTATGAAAAAGGCTAAAATACTTTCTGGCGTATTATTACTGTGCTTTTCGTCCCCATTAATTTCTCAGGCTGCGACACTGGACGTACGTGGTGGATATCGTA	nanC
